# Simulation-based education and sustainability: creating a bridge to action

**DOI:** 10.1186/s41077-025-00354-2

**Published:** 2025-05-08

**Authors:** Katherine Mitchell, Robyn Canham, Katie Hughes, Victoria Ruth Tallentire

**Affiliations:** Medical Education Directorate, NHS Lothian, Edinburgh, Scotland

**Keywords:** Simulation-based education, Education for sustainable healthcare, Carbon costing, Carbon footprint, Environmental impact, Quality improvement, Translational simulation, Sustainable simulation toolkit

## Abstract

**Background:**

In light of growing environmental concerns, this article examines the often-overlooked environmental impact of simulation-based education (SBE) within healthcare. We position simulation professionals as agents for environmentally sustainable change and seek to empower achievable, meaningful, measurable action. As a high-value yet resource-intensive pedagogical tool, SBE frequently relies on energy-intensive technologies and single-use materials that contribute to carbon emissions and waste. This article explores the environmental impact of SBE, detailing how it contributes to the healthcare sector's impact on the triple planetary crisis; climate change, pollution, and biodiversity loss.

**Main messages:**

Within the simulation community, we have observed a high level of motivation to respond to the triple planetary crisis and make sustainable change. However, there is limited information available to simulation educators about practical changes that can be made. We have responded with an article that can help move from rhetoric to action, from inertia to empowerment.

Understanding the environmental impact of simulation activities provides a useful starting point. We explain how to estimate a carbon footprint for SBE and how this relates to its wider environmental impact. Recognising the urgent need for change, we then present a comprehensive toolkit of practical strategies that can improve the environmental impact of SBE.

Part one of our toolkit focuses on resource management, waste reduction and efficient session delivery. In part two, we highlight how principles of sustainable healthcare can be incorporated into scenario design and local strategy. This more holistic approach shows how SBE can be leveraged beyond immediate educational goals to foster sustainable practice in healthcare.

We present evidence for our toolkit, detailing the principles and frameworks on which the suggestions are based. Additionally, we discuss how change can be measured and what risks educators should be aware of.

**Conclusion:**

By embedding sustainability into SBE, educators can not only mitigate their own environmental impact but also model sustainable healthcare practices for learners. Through these steps, the simulation community can play a pivotal role in addressing healthcare’s environmental impact and contribute to a healthier planet.

**Graphical Abstract:**

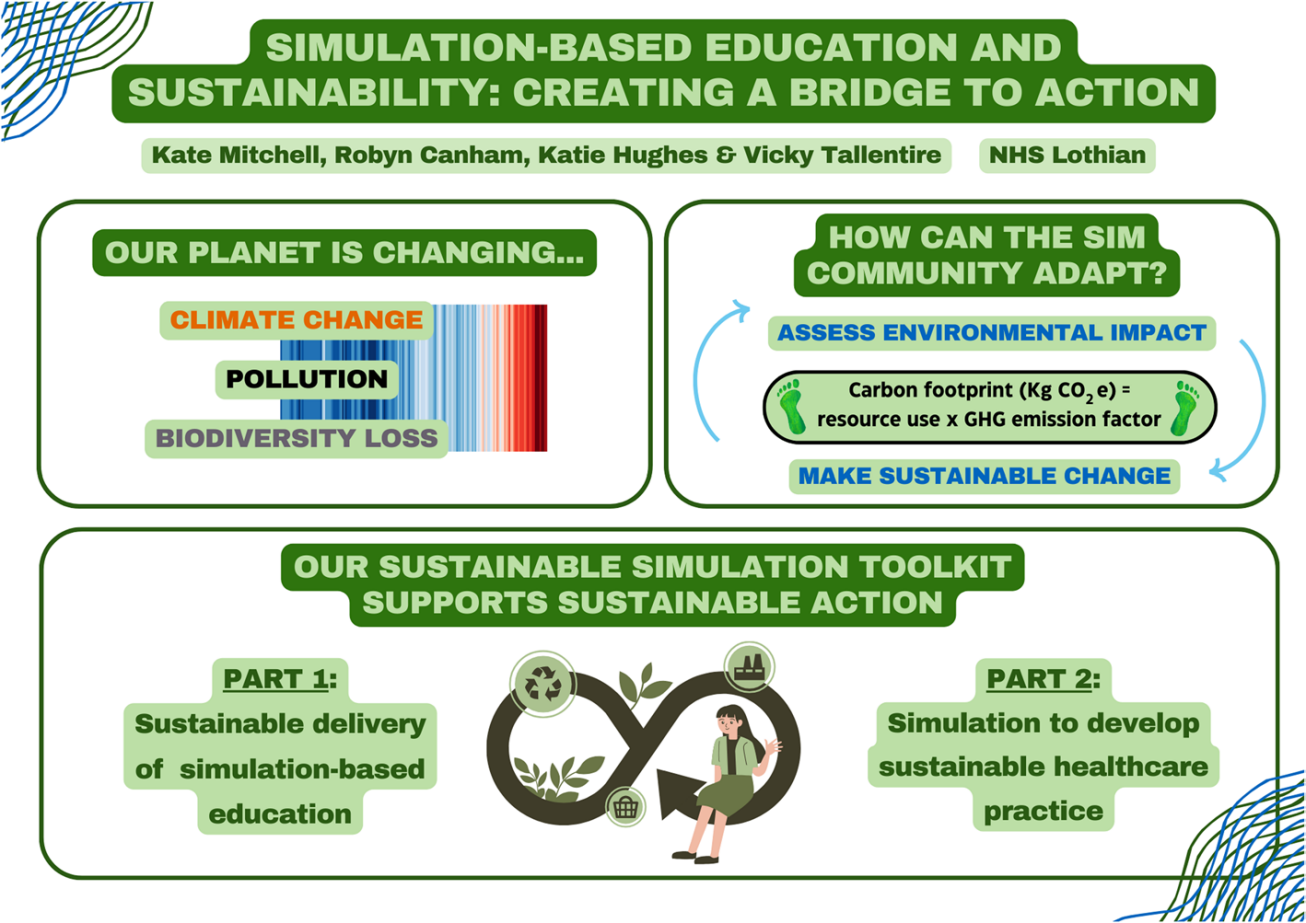

## Our changing planet and opportunities to adapt simulation-based education

### The start of our story

During a recent faculty meeting, one of our experienced simulation facilitators asked:"How often do we consider the environmental impact of our simulation sessions?"The room fell silent. Focused on developing clinical practice, we had not thought enough about the energy used to run equipment or the materials thrown away at the end of the day. This brief reflection sparked a conversation about the hidden environmental impact of simulation and left the group with a profound realisation: Simulation is saving lives but at an unseen cost to the planet.

This scenario is a microcosm of a larger issue facing health professions education today. As educators, we prepare students to tackle multiple health challenges. These include health challenges resulting from climate change—the greatest global threat to health in the twenty-first century [1]. But, do we overlook simulation-based education’s (SBE) contribution to climate change, as we teach students to manage the consequences of this phenomenon? Here we explore not only the environmental impact of SBE, but what we can do to improve this, “*in our hands now lies not only our own future, but that of all other living creatures with whom we share the earth*” [2].

### The triple planetary crisis

The triple planetary crisis, comprising climate change, pollution, and biodiversity loss, is one of the most significant challenges facing humanity today [3]. Rising global temperatures, increasing pollution levels, and a sharp decline in biodiversity are converging to create environmental conditions that threaten human health, ecosystems, and the sustainability of life on Earth [3, 4]. The healthcare sector, which is responsible for around 4.4% of global greenhouse gas emissions, is a key contributor to this crisis [1, 5, 6]. These emissions result not only from direct healthcare activities, but also from the broader processes and resources involved in healthcare delivery, such as health professions education.

Health professions education, including SBE, plays an important but often overlooked role in contributing to healthcare’s environmental impact [7]. Simulation, while a highly valued pedagogical tool, can be resource-intensive, relying on energy-consuming technologies and disposable materials which contribute to environmental degradation [8]. Therefore, it is imperative for simulation educators to critically assess and minimise the environmental impact of their practice.

### The current position

Despite the necessity to act decisively and save our planet, a sense of inertia has been observed amongst many healthcare professionals [9]. Ihsan et al. propose that lack of understanding contributes to this inertia; highlighting poor understanding not just of healthcare’s environmental impact but (more importantly) what health professionals can do to improve it [9]. This lack of understanding is not surprising given the sporadic inclusion of climate change and its relationship to health in health professions curricula [10–12]. Additionally, we have observed a perception that the triple planetary crisis is too vast and complex a problem to tackle on an individual, or even an institutional, level. This highlights the clear need for educators to move beyond awareness-raising towards concrete strategies that address sustainability within simulation and across health professions education.

A recent questionnaire distributed via multiple global simulation educational groups found that supply reuse has been adopted as a common sustainability practice in the United States and internationally. However, only 40% of respondents had a written sustainability plan and over 50% stated that carbon neutrality was not a consideration for their simulation centre [8].

### Moving from rhetoric to action

Our article responds to the call in the Association for Medical Education in Europe’s (AMEE) Consensus Statement on Planetary Health and Education for Sustainable Healthcare [13], which urges health professions educators to shift from rhetoric to action. Embedding sustainability into the core of SBE also aligns with current Association for Simulated Practice in Healthcare (ASPiH) recommendations, which set out the need for simulation practice to consider sustainability in learning outcomes and in managing resource use [14].

This article aims to provide practical advice to assist simulation educators in their understanding of, and engagement with, sustainability, regardless of the context of their SBE. The article comprises three parts; the first focuses on calculation of the carbon cost of SBE, the second on delivering SBE more sustainably (Toolkit Part 1) and the third on harnessing the power of SBE as a pedagogical tool to develop sustainable healthcare practice (Toolkit Part 2).

Each suggestion is underpinned by widely recognised principles such as the United Nations Sustainable Development Goals (UNSDG) [15], the Planetary Boundaries framework [16] and the circular economy system [17, 18]. Additionally, standards specific to clinical education and SBE [13, 14, 19] have been used to guide the suggestions made.

In the clinical setting, similar toolkits such as the GreenED framework have been shown to reduce environmental impact and financial spend [20]. Our hope in producing this article is to catalyse a shift from inertia to empowerment in the field of SBE, equipping educators with the knowledge, motivation and tools to make meaningful contributions to planetary health.

## Understanding the environmental impact of simulation-based education

### Carbon footprint

**Fig. 1:**
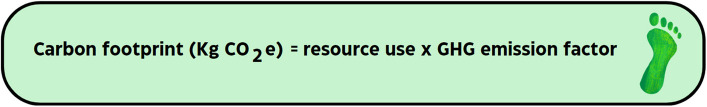
Formula for calculating the carbon footprint to quantify environmental impact

SBE often involves energy-intensive technologies, such as high-fidelity simulators, and single-use plastic consumables. Quantifying the environmental impact of SBE is challenging and there remains a lack of meaningful data in this domain [21]. Carbon cost, or ‘footprint’, is the most frequently utilised way of quantifying environmental impact. Carbon footprint refers to the total amount of greenhouse gases (GHGs) emitted by an activity or product and is expressed as carbon dioxide equivalent (CO2e) [22] (Fig. 1).

### Broader environmental considerations

It is important to acknowledge the environmental impact of SBE reaches far beyond emission of GHGs. There are other negative environmental consequences such as deforestation and a loss of biodiversity during the production of paper-based products, and reduced air quality because of particulates released by fossil fuel-powered vehicles carrying simulation equipment. Such impacts are more difficult to quantify, but by focusing on reducing the carbon footprint of simulation-based activities it is likely a ripple effect will lessen other negative consequences [23, 24].

### Calculating carbon footprint in simulation-based education

While determining the overall carbon footprint of an institution can be complex, a structured approach to evaluation can guide educators towards making more sustainable choices and assessing the impact of any changes made.

The Greenhouse Gas Protocol provides widely used standards for the accounting of greenhouse gas emissions [25–27], It classifies emissions into three different categories, or “scopes”. Scope 1 emissions are direct emissions from sources owned or operated by the institution. Scope 2 emissions are emissions released in the production or use of purchased energy. Scope 3 emissions are the indirect emissions that occur because of an institution’s activities. These include emissions that occur at other organisations due to products or services provided to an institution, or emissions that are released at other organisations due to outputs from an institution, for example, waste processing [28]. When considering healthcare and healthcare-related activities, scope 3 emissions tend to form the most significant contribution to an organisation’s carbon footprint [21].

To calculate carbon footprint, the GHG emission factors for a resource need to be identified. GHG emission factors for many of the activities carried out, and products used, in the delivery of SBE are freely available from a variety of sources. Government and institution sources for GHG emission factors are available online, including those published by the UK government [26] and the United States Environmental Protection Agency [27]. The Centre for Sustainable Healthcare's ‘measuring environmental impact’ calculator also provides a useful summary of GHG emission factors related to healthcare activities [23].

Table 1, adapted from a carbon footprint assessment of NHS England activities, highlights emissions associated with SBE and gives examples of GHG emission factors that can be used to calculate the carbon footprint of SBE. For some resources, such as medical equipment, the GHG emission factor of an individual item is not known. It is therefore common practice to use the overall financial spend when calculating carbon footprint for such resources [23].

**Table 1:** Key areas of resource use in the delivery of simulation-based education^a^ [23, 26, 29]

	**Example of resource use**	**Example GHG emission factor**^a^
**Scope 1:** Direct emissions from sources owned or controlled by the institution	Institution owned and leased vehicle use	0.21656 kgCO_2_e/km (car travel)
	Emissions from fossil fuel use on site e.g. powering simulators	Electricity use (UK): 0.2913 kgCO_2_e/kWh Natural gas: 0.21451 kgCO_2_e/kWh
**Scope 2:** Indirect emissions released from the production or use of purchased energy	Emissions associated with purchased energy supplies	No data found
**Scope 3:** Indirect emissions that occur because of the intuition’s activities	Water consumption	366.6 kgCO_2_e/million litres
	Sewage processing	0.361 kgCO_2_e/£
	Medical equipment, e.g. cannulas; phlebotomy equipment; fluid bags	0.3 kgCO_2_e/£
	Medical instruments	0.41 kgCO_2_e/£
	Disposable gloves	0.026 kgCO_2_e/item
	Single use apron	0.065 kgCO_2_e/item
	Food and catering	0.64 kgCO_2_e/£
	Office equipment	0.53 kgCO_2_e/£
	Clinical waste processing	569 kgCO_2_e/tonne
	Domestic waste processing	172 kgCO_2_e/tonne
**Non-protocol**	Participant travel	Bus: 0.12721 kgCO_2_e/passenger.km Car: 0.21656 kgCO_2_e/km Train: 0.04282 kgCO_2_e/passenger.km Regular taxi: 0.18508 kgCO_2_e/passenger.km

^a^GHG emission factors displayed in the table are taken from UK and NHS England data. They can be used as a guide outside of this context, but more accurate GHG emission factors may be available from alternative government and institution sources

## A bridge to action

### The toolkit—part 1: practical steps to reduce carbon footprint

Reducing the carbon footprint of SBE does not require a complete overhaul of existing systems, but can be achieved through practical, incremental changes and robust quality improvement (QI) work. Table 2 outlines the first part of our toolkit which focuses on changes that can be made in the delivery of SBE and expands on the principles described by Chanchlani et al. [21]. This part of the toolkit is informed by several frameworks and principles, as described below.

**Table 2:** Toolkit part 1—sustainable changes to the delivery of SBE

Area of resource use (environmental aspect)	Suggested changes	Supporting frameworks	Potential impacts and how to measure these	Potential risks and mitigation
Supports ISO 14001 clause 6: planning [36]	Supports ISO 14001 clause 8: operations		Supports ISO 14001 clause 9: performance and clause 10: improvement	
*Energy consumption*	• Turn off simulators and other equipment when not in use • Use energy efficient lighting/products • Use renewable energy sources where possible • Ensure efficient booking so that sessions are not run multiple times with low participant numbers	• The Paris Agreement (2016) • The Planetary Boundaries Framework (2009) • UNSDG 7–Affordable clean energy–requires increased energy efficiency and growth of modern renewable energy sources (2015) • The Circular Economy System (2017) • Standardised Carbon Emissions Reporting for Higher and Further Education (SCEF) (2024)	• Reduces unnecessary energy consumption and associated GHG emissions • Reduced pollution from burning fossil fuels • Financial savings • *Energy consumption can be measured using meters or energy-monitoring smart plugs. Reduction in GHG emissions and financial spend can be extrapolated from this* • More efficient use of simulation team time • *Simulation sessions delivered, participant numbers and faculty numbers can be monitored*	• The financial cost of different energy providers must be monitored • Quality of learning should be evaluated if changes are made to simulation programme delivery
*Water consumption*	• Reduce water consumption where possible. This could be through actions such as minimising linen use	• The Planetary Boundaries Framework (2009) • UNSDG 6–Clean water and sanitation–requires acceleration of the implementation of integrated water resources management (2015) • The Lancet Commission on Planetary Health (2015)	• Preservation of water as a natural resource • Reduced GHG emissions associated with water treatment • *It may be possible to monitor water usage using meters* • *The use of products that require laundering can be monitored and reduction in GHG emissions extrapolated from this*	• Cleanliness and sterility must be monitored (where relevant)
*Material use (presented in accordance with the 9r framework)*	• Refuse-consider materials/products procured—are they all necessary? • Rethink-pool equipment with neighbouring simulation centres • Reduce-consider how simulation is delivered-can intended learning outcomes be met with lower fidelity simulation? Could virtual reality or augmented reality simulation support your simulation centre to improve environmental impact? - If possible, use out of date equipment that might otherwise be thrown away • Re-use-encourage participants to bring reusable cups/water bottles for personal use - Reuse equipment when possible—create posters to explain which items should be kept and which disposed of • Repair-repair items when possible • Refurbish, remanufacture and repurpose-opt for suppliers that demonstrate robust environmental credentials; e.g. use of non-oil derived polymers, willingness to take back used simulators for refurbishing/re-purposing and have a clear commitment to zero waste operations - Procure previously used/repurposed equipment when possible • Recycle-ensure used materials are recycled where possible • Recover-opt for biodegradable or recyclable materials where possible	• The Circular Economy System (2017) • The 9R Framework (2017) • UNSDG 12 – Ensure sustainable consumption and production patterns (2015)	• Fewer natural resources required to create new products • Reduced GHG emissions from production of new products • Reduced waste • Financial savings from both procurement and waste disposal • *Number of a particular item used by the simulation centre can be monitored. From this changes in associated GHG emission can be calculated* • *It might be practical to focus on one or two items initially and gradually build on this work*	• GHG emissions and financial cost associated with transport must be balanced against those associated with procurement of equipment for individual centres • Financial cost must be monitored if opting for different suppliers • Consider the potential unintended consequences to learning and the future clinical practice of learners when changing to lower fidelity simulation methods or virtual or augmented reality-based simulation. Consider regular evaluation of changes made to scenario design and delivery to ensure it is still meeting the needs of learners
*Food consumption*	• Opt for foods with a lower carbon footprint—these will generally be vegetarian/plant based food options	• The Lancet Commission on Planetary Health (2015)	• Reduced GHG emissions • Potential health benefits of a vegetarian/plant based diet • Reduced cost • *Food purchased and any food waste generated can be monitored. From this information associated GHG emissions and financial spend can be calculated*	• Food options might be less appealing therefore generating more food waste • Financial cost needs to be monitored
*Waste disposal*	• Reuse items when possible to minimise waste • Re-home unwanted items, if this is not possible consider if the item could be repurposed • Segregate waste appropriately and recycle where possible	• The 9R Framework (2017) • The Circular Economy System (2017) • SCEF (2024)	• Reduced GHG emissions • Financial savings • *Amount of waste in each ‘stream’ can be measured and changes in GHG emissions and financial spend extrapolated from this*	• Waste should be checked to ensure any hazardous waste, such as clinical waste/sharps, is appropriately disposed of
*Transport*	• Explore virtual simulation options • Consider work from home options for staff if appropriate • Promote the use of active travel, public transport and carpooling • Consider the location of your simulation sessions in relation to location of participants-could the session be delivered outside of the simulation centre by adjusting scenarios/equipment?	• The Paris Agreement (2016) • The Planetary Boundaries Framework (2009) • SCEF (2024)	• Decreases travel-related emissions, particularly for geographically dispersed simulation team/learners • *Mode and distance travelled by participants and faculty can be measured* • Varying locations and increased working from home may be seen as positive changes by staff and could improve recruitment and retention	• Ensure accessibility is maintained for learners and staff. Consider surveying staff and learners after changes to location of simulation sessions/place of work are made • Potential impacts on recruitment and retention of staff could be positive or negative. Risks could include impacts on ability to balance the delivery of simulation sessions alongside clinical roles if sessions are moved away from central clinical sites

### Founding frameworks and principles

The Paris Agreement unites the international community in a commitment to reduce GHG emissions, with the aim of limiting global temperature rise to 1.5 degrees Celsius above pre-industrial levels and thus reducing the risks and impacts of climate change [30, 31]. Planetary Boundaries [16] comprise nine different categories of change to our planet: climate change, novel entities, stratospheric ozone depletion, atmospheric ozone depletion, ocean acidification, biogeochemical flows, freshwater change, land-system change, and biosphere integrity. For each category a boundary has been set; within this boundary there is a safe operating space for human civilisation while moving beyond the boundary risks irreversible environmental change. Six of the nine boundaries, including climate change, have been transgressed, suggesting that GHG emissions must urgently be reduced [32].

The circular economy system describes using materials more effectively and for longer periods of time [17, 18]. This relies on transition to renewable energy and materials—improving sustainability by reducing climate change, pollution and biodiversity loss. Because many of the resources on our planet are finite, losing material to landfill or incineration is not a long-term option. Moving towards a circular economy helps address this and reduce consumption of raw materials [33].

The 9R framework is thought to enable progression from a linear to a circular economy [18, 34]. The framework describes the use and re-use of materials at their highest value, demonstrating that smarter use of materials (refusing, rethinking and reducing) gives the greatest gain, followed by extending the lifespan of a product and its parts (reusing, repairing, refurbishing, remanufacturing and repurposing) [18]. In our toolkit, the suggestions relating to material use are set out in accordance with the 9R framework (Table 2).

The United Nations Sustainable Development Goals (UNSDG) recognise that ending poverty must go hand-in-hand with health and education, tackling climate change and preserving the natural world [15] Five of the 17 UNSDG goals are particularly pertinent to our toolkit: good health and wellbeing, quality education, clean water and sanitation, affordable and clean energy and responsible production and consumption.

Almost ten years ago, The Lancet commission on Planetary Health published a report concluding *“…the continuing degradation of natural systems threatens to reverse the health gains seen over the last century”* [35]. The suggestions in our toolkit are built around some of the recommendations made by the commission including; reducing food waste, eating healthy diets with a low environmental impact and using water more efficiently. The 2023 assessment of planetary boundaries [32] shows that 10 years on from the commission’s report these recommendations still need to be addressed; our toolkit enables change with practical suggestions for simulation educators.

### Using the toolkit in individual contexts and simulation centres

To ensure proposed changes have the desired action (improved sustainability of SBE), we suggest how the impact of these changes can be measured. We also identify potential risks that should be monitored when implementing the toolkit, and steps to mitigate these. Although any change has risks and may bring unintended consequences, we believe it is critical to consider that perhaps the greatest risk lies in not making sustainable change and continuing to exploit the planet's natural resources [16].

Environmental management systems (EMS) can assist organisations in monitoring and managing their impact on the environment. The International Organization for Standardization (ISO) sets out an internationally recognised standard for creation and use of an EMS in ISO 14001 [36]. ISO 14001 can be applied to any organisation, but each must use the framework to create an EMS suitable for their individual needs. The plan-do-check-act cycle (PDCA) is then applied across different parts or ‘clauses’ of the EMS to ensure continuous improvement. Our toolkit is specific to SBE and so alone, or in combination with a generic tool such as ISO 14001, it can be used to support simulation centres to make their operations more sustainable. For centres already working towards ISO 14001 certification, or planning to do so, we have mapped the ISO 14001 clauses across our toolkit (Tables 2 and 4). Additionally, reporting frameworks can help organisations quantify their environmental impact; thus enabling them to better understand their contribution to the triple planetary crisis and to respond effectively. Standardised Carbon Emissions Reporting for Higher and Further Education (SCEF) [19] identifies several emissions categories that can be measured by educational institutions (e.g. emissions from waste generated in operations), allowing them to focus on where to make improvements.

### An example of sustainable change

Consider a twice-weekly simulation session for 10 final-year medical students, focusing on assessing an acutely unwell patient. Each session comprises three scenarios, and during each scenario, participants would be expected to insert a cannula and administer intravenous fluids. The primary learning outcome of the session is for participants to understand and demonstrate a systematic patient assessment. If 60 sessions run per year, a total of 180 cannulas would be used over the academic year.

Considering the 9R framework, we can identify changes that should make the session more environmentally sustainable [18, 34]. The number of cannulas procured for this session could be reduced (R3) by using supplies from clinical areas that have passed the expiry date. Another strategy would be reducing the total number of cannulas used in the sessions. This can be achieved by leaving a cannula in situ on a simulation trainer and asking participants to verbally communicate to embedded faculty within the scenario their intention to cannulate the patient (rather than actually performing the procedure). From an environmental perspective, this would result in using fewer natural resources during the production of the cannulas and reduced GHG emissions associated with their production and disposal.

**Fig. 2:**
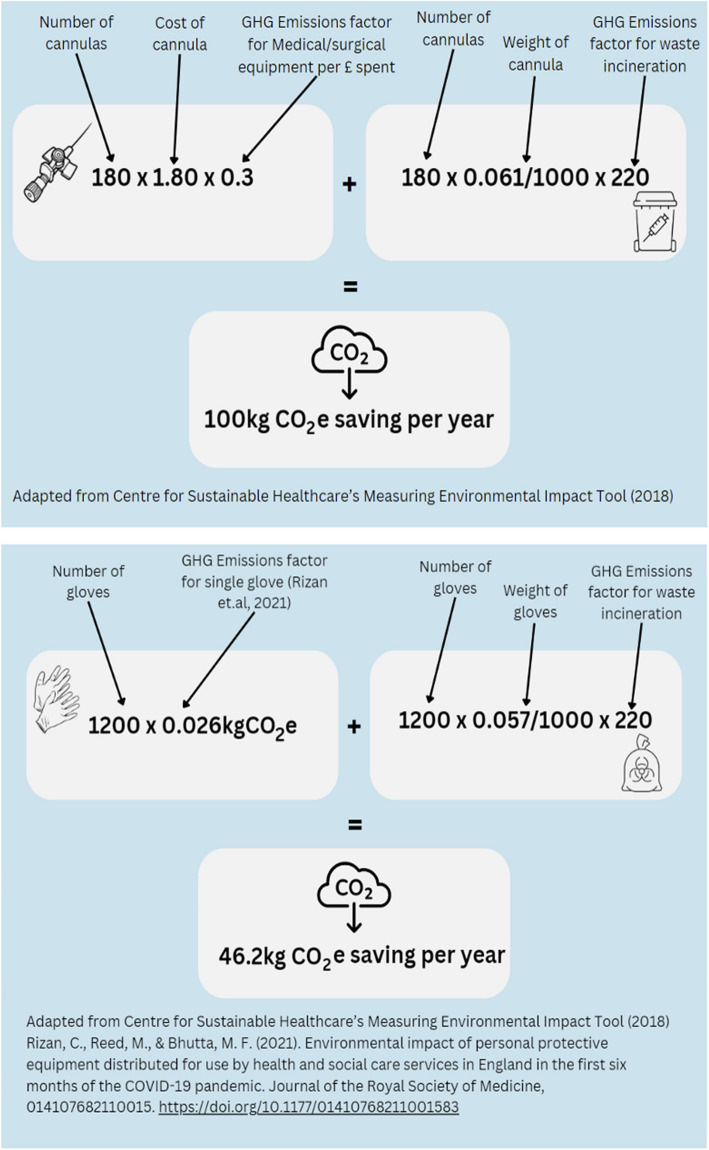
Worked example of calculating carbon saving by reducing the use of cannulas and disposable gloves in a simulation session

Another option to reduce resources used during the scenario would be advising participants they are not required to wear gloves, even if performing a task within the simulation that would require them to wear gloves in clinical practice. Assuming that previously, each participant was wearing one pair of gloves during the session, 1200 fewer disposable gloves would be used and sent for incineration per year. Figure 2 demonstrates how to estimate the kgCO2e saved by removing single-use gloves and leaving a single cannula in situ within the scenarios.

With any alteration to scenario design, it is important to consider the pedagogical impact of any changes made. Using the example above, the primary goal of the scenario is for participants to demonstrate a systematic approach to patient assessment. In this case, it could be argued that neither of the altered components is crucial to the participants achieving the intended learning outcomes, provided they can correctly identify the need to cannulate the patient. If, however, one of the intended learning outcomes was for participants to demonstrate safe and sterile procedural skills (such as intravenous cannulation), removing the need to wear gloves and pre-inserting a cannula into a simulation trainer may have potential impacts on their ability to achieve this learning objective. It could also influence safe performance in clinical practice, where there is a requirement for practical procedures to be performed in line with local personal protective equipment (PPE) policy. Although there is currently no evidence to support this theoretical concern, the potential pedagogical costs to learners requires close observation.

When considering PPE, there are risks that it is overused as well as underused in both clinical and simulation practice [37]. It is therefore important that we consider carefully when PPE adds value and when it worsens the environmental impact of SBE without adding educational value. It can be challenging to decide when the environmental benefits of altering established educational practices outweigh hypothetical risks. Regular evaluation of participants’ learning and their clinical practice should allow organisations to address any unintended consequences from their desire to improve the environmental sustainability of SBE.

### The toolkit—part 2: simulation to drive environmental sustainability in healthcare education

We suggest SBE has the potential to mitigate the triple planetary crisis beyond direct changes made to delivery and organisation of sessions. Although SBE is traditionally used to improve knowledge and measure performance, it can also be a powerful tool when used in the realm of quality improvement and systems change work [38, 39]. In this section, we discuss how simulation can be used as a powerful and effective instrument to promote sustainable practice by encouraging innovation, testing ideas in a risk-free environment, and embedding sustainability as a core value in healthcare education.

### Simulation to develop sustainable practice

Simulation is increasingly used in QI initiatives, where it provides a space for testing and refining clinical practices [38]. Similarly, it can be harnessed to address the environmental impact of healthcare systems. The concept of"work as done"—understanding how tasks are actually carried out, as opposed to"work as imagined"—is central to the role of simulation in QI [39]. In this context, simulation can be used to explore how sustainable practices might function in the real world, revealing potential barriers and solutions to incorporating these practices into clinical workflows.

For example, a simulation scenario could focus on reducing single-use plastic during a routine procedure, such as central line insertion. Participants might be asked to explore alternatives to disposable items, reconsider waste management practices, or evaluate the necessity of the procedure itself. Participants could be supported in their thinking by the 9R Framework and other relevant principles (Table 2). The simulated environment allows for creativity and collaboration, enabling participants to think critically about the environmental impact of their clinical decisions. Simulation in this context is not only about teaching clinical skills but also about instilling a mindset of sustainability [40, 41].

This approach mirrors the increasing utility of translational simulation, where the goal is to diagnose and address safety or performance issues in healthcare delivery [42]. However, by reframing simulation as a tool for diagnosing and addressing sustainability issues, educators can focus on minimising the environmental footprint of clinical practice. This approach enables healthcare professionals to ‘test drive’ environmentally friendly solutions, making it easier to implement these changes in real-world settings.

### Embedding environmental sustainability in the design of simulation-based education

SBE offers a unique opportunity to embed the principles of environmental sustainability directly into healthcare training. Just as simulation has been used to promote equity, diversity, and inclusion (EDI) in healthcare education [43], it can also be a platform for changing attitudes towards sustainability. The immersive, experiential nature of simulation allows participants to engage with complex topics like environmental sustainability in a meaningful way, helping to connect abstract concepts to practical actions. For example, in a debriefing following a simulation exercise focused on reducing waste, educators can encourage reflective discussions on the ethical implications of environmental harm caused by healthcare. Such conversations can help shift mindsets, turning sustainability from a peripheral concern into a core value that guides clinical decision-making.

**Fig. 3:**
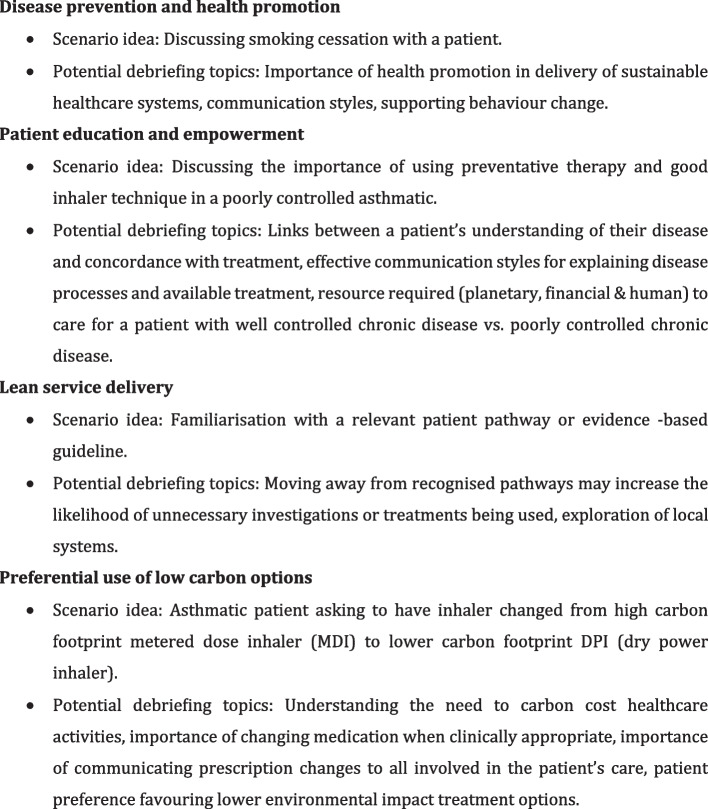
Scenario and debriefing ideas that incorporate principles of sustainable healthcare into simulation design

To fully integrate sustainability into SBE, educators should critically appraise their existing curricula and explore how environmental issues can be woven into intended learning outcomes. Mortimer describes four principles of sustainable healthcare: disease prevention, patient education and empowerment, lean service delivery and low-carbon alternatives [44]. These principles should be applied to simulation scenarios, we offer examples of how this could be done in Fig. 3. Many of the suggested scenarios will present the opportunity to debrief around a wide variety of intended learning outcomes (ILOs). We suggest scenarios should always include, either explicitly or implicitly, consideration of sustainable healthcare principles.

Consider a scenario where students must decide between different treatment options based on their environmental impact—as suggested in Fig. 3 in relation to inhalers. Such a scenario could lead to discussions around how we balance clinical efficacy with sustainability and financial burden, trying to identify interventions that offer an improvement in all three of these domains. By embedding sustainability into the ILOs of SBE, educators can foster the culture of environmental responsibility among current and future healthcare professionals.

### Changing attitudes: a tool for hearts and minds

SBE facilitates experiential learning that can deepen understanding and commitment to sustainability. When learners experience first-hand how sustainable practices can be incorporated into clinical care, they are more likely to carry those lessons into their future practice. By creating scenarios that highlight the environmental consequences of clinical actions, SBE can help shift attitudes and promote long-term behavioural change.

The second part of our toolkit summarises the ideas above and focuses on improving environmental sustainability through scenario design and development of a local sustainable simulation strategy (Table 3). Although the need for health professionals to practice sustainably has been highlighted by regulatory bodies [45], the principles of sustainable healthcare are not yet widely included in health professions curricula [10–12]. Our toolkit offers suggestions that empower simulation educators to address this gap.

**Table 3:** Toolkit part 2—improving sustainability through scenario design and local strategy

Key area of focus	Suggested changes	Supporting frameworks	Potential impacts *and how to measure these*	Potential risks and mitigation
*Scenario design* Supports ISO 14001 clause 6: planning	• Integrate principles of sustainable healthcare into existing scenarios by adapting intended learning outcomes and/or debriefing • Try designing new scenarios with sustainable healthcare at their core	• Principles of sustainable healthcare (Mortimer) • ASPiH standards (2023) • Generic professional capabilities framework (GMC)	• Raises awareness of sustainable healthcare • Demonstrates how principles of sustainable healthcare can be applied to practice • Encourages learners to view their practice with sustainable healthcare systems in mind	• ‘Overburdening’ learners with too many ILOs that could potentially detract from learning other content • Evaluation of adapted and newly developed sessions will help ensure high quality education
*Sustainability prioritized by the leadership team and included in local simulation strategy* Supports ISO Clause 5: Leadership and clause 7: support	• Review local simulation strategy-ensure sustainability is prioritised; key actions have been identified and assigned to individuals or groups within the leadership team and wider staff group • Appoint a lead for sustainability in your simulation centre—the sustainability lead should work closely with and/or be a part of the simulation centres leadership team • Ensure job plan time is allocated to these roles	• ASPiH standards (2023)	• Can ensure a planned, organised and carefully thought through approach to sustainable simulation • *Monitor this using QI methodology* • Improved engagement with sustainability can improve staff retention and recruitment • *Survey staff to find out if prioritizing sustainability has affected their decision to work for the organisation*	• Avoid overburdening staff by ensuring there is adequate time, training and resources to support staff in their sustainability roles
*Share ideas and learning* Supports ISO clause 7: support	• Forge links and join networks that allow the sharing of sustainability work and resources (e.g. EAUC and sustainability partnership in UK)	• ASPiH standards (2023)	• Allows institutions and individuals to learn from each other	• Strategies that are effective in one setting may not be effective in all. Ensure changes are measured and monitored using QI methodology
*Faculty development* Supports ISO clause 7: support	• Provide training, onboarding opportunities, drop-ins, FAQ sessions and meta-debrief clubs that focus on environmentally sustainable simulation	• ASPiH standards (2023)	• Creates an empowered and engaged faculty group who can develop collectively and incorporate environmental sustainability into their simulation practice • *Survey staff to find out what they have learnt about sustainability and how they apply this to their role*	• Faculty development focused on sustainability could divert resources away from other areas. The centre should review staff training to ensure training resources are used to their greatest advantage

Both parts of the toolkit could also be used as a checklist with QI methodology employed to test and evaluate the changes. As mentioned above, this approach is recommended in the ISO 14001 framework [36] which suggests using the PDCA when monitoring environmental impact.

## Overcoming challenges: upskilling faculty and creating safe spaces

One of the challenges of incorporating sustainability into SBE is that faculty members may feel inadequately prepared to lead these discussions. Educators may worry their own knowledge of environmental sustainability is insufficient or that they may not be able to answer all their learners'questions. However, this challenge can be addressed through faculty development focused on environmental sustainability, similar to the successful approaches used in upskilling faculty to incorporate EDI into SBE [43].

Furthermore, simulation educators already possess core transferable skills, such as creating supportive learning environments and guiding reflective practice [46]. These core skills can be enhanced using meta-debrief clubs focusing on sustainable healthcare. Such activities provide a ‘safe container’ where educators can feel comfortable sharing and exploring new ideas together [47, 48]. By fostering curiosity and collaboration in these psychologically safe spaces, educators can empower each other, and in turn their learners, to explore innovative solutions to the environmental challenges facing healthcare.

## Conclusions

This article has highlighted the significant environmental challenges posed by the triple planetary crisis and the role that healthcare, particularly SBE, plays within it. While the carbon footprint of healthcare and simulation is often underestimated, there is an urgent need for educators and practitioners to recognise the impact of their activities. More importantly, the simulation community has the potential to be a powerful force for positive change by adopting more sustainable practices and embedding environmental awareness into their curricula and activities. Our toolkit provides a practical, evidence-based guide to aid this process for individual simulation centres and programmes. This approach has been used successfully in the clinical setting and as our work progresses, we will be further testing and developing our toolkit, expanding the evidence base for its use.

SBE can serve as both a contributor to, and a solution, for the environmental crisis. The challenge lies in transforming simulation from a resource-intensive activity into one that models and promotes sustainable healthcare practices. By using innovative pedagogical tools such translational simulation approaches, educators can help shift the focus towards sustainability, not only in education but also in clinical practice.

We call on the simulation community to critically evaluate their practices and ask what more can be done. Small, incremental changes, such as rethinking resource usage, minimising waste, and integrating sustainability into learning outcomes, can have a significant cumulative effect. It is essential for educators to challenge the status quo, foster collaborative environments, and empower both students and clinicians to embrace sustainable practices. In doing so, SBE can become a catalyst for broader changes in healthcare systems, ultimately contributing to a healthier planet.

## Data Availability

No datasets were generated or analysed during the current study.
